# Demography and Behaviour of *Teinopodagrion oscillans* (Odonata: Megapodagrionidae) in a Protected Area of the Colombian Andean Region

**DOI:** 10.3390/insects15020125

**Published:** 2024-02-09

**Authors:** Fredy Palacino-Rodríguez, Diego Andres Palacino, Andrea Penagos Arevalo, Adolfo Cordero-Rivera

**Affiliations:** 1 Etology Section, Faculty of Sciences, Republic University, Montevideo 11200, Uruguay; 2 Research Group on Odonata and Other Arthropods in Colombia and the Neotropics (GINOCO), Sesquilé 251057, Colombia; dblazer155@gmail.com (D.A.P.);; 3 ECOEVO Lab, E. E. Forestal, Campus Universitario A Xunqueira s/n, Universidade de Vigo, 36005 Pontevedra, Spain; adolfo.cordero@uvigo.gal

**Keywords:** damselfly, dragonfly, aquatic insects, mark-recapture, Neotropics, Zygoptera

## Abstract

**Simple Summary:**

Basic information on the ecology and behaviour of many aquatic insects is lacking because of the absence of studies. Here, we investigated whether demographic aspects such as survival, sex ratio, and population size are different between sexes and individuals of different ages of *Teinopodagrion oscillans*, a damselfly species endemic to the Andean region, living in a protected area. We also recorded the behaviour of this species and the area frequented by the adults near the water. Survival, recapture, and lifespan (14.1 ± 0.59 days) were similar for both sexes and all age groups. Mature males were larger, while the distance from the water where animals perched was similar for all individuals. Weather variations affected the demography of this population in a significant way. Individuals perch on any available support (≤0.1–12 m high) and show high fidelity to their perch site, with mature males making short flights from the perch to intercept other individuals and to hunt prey. The tandem position was formed on macrophytes, and females lay eggs by submersion of their abdomen. Our results provide crucial information for short- and long-term conservation decisions of the biodiversity in ecosystems of the Andean region.

**Abstract:**

The demography and behaviour of *Teinopodagrion oscillans* was studied in a protected area in the Andean region of Colombia. Adult damselflies were individually marked, and using their recapture histories, we estimated survival, longevity, sex ratio, and population size using Cormack-Jolly-Seber models. Other aspects of their behaviour were recorded. Survival, recapture, and lifespan (14.1 ± 0.59 days) were similar for both sexes and all age groups. Mature males were larger, and the distance from the water was similar for all individuals. The most supported model was the time-dependent model for survival and recapture. This suggests that weather variations affect the demography of this population in a significant way. Individuals exhibited high fidelity to their site perch, perching with open wings near water on a variety of perches. Mature males make short flights from the perch to intercept conspecific and interspecific males and to hunt prey. The tandem position was formed on macrophytes, and then the pair flew away. Oviposition lasted for 11.23 min on average, with the females ovipositing by abdomen submersion. Our results offer insights into the demographic characteristics and behaviour of this species, providing crucial information for the short- and long-term, from the demography of one species to the conservation of ecosystems of the Andean region.

## 1. Introduction

Research in animal demography has generated a substantial body of literature, allowing a good understanding of how populations change over time [1,2,3]. Historically, demographic information has been used in studies focusing on the context of stable population theory [4,5], life tables [6], and population biology [7]. However, an emerging sub-discipline of demography, bio-demography, suggests going further, using the demographic information to understand and explain the underlying biological and ecological mechanisms acting on the populations [8]. The current development of approaches based on maximum likelihood models allows for the estimation of demographic parameters using recapture histories of marked individuals because these methods consider the variable detection rate of individuals to estimate survival rates and population sizes [9,10]. 

Although mark-recapture and maximum likelihood methods have been widely used in organisms such as damselflies (reviewed by [11]), research on Neotropical species is just beginning [12,13,14,15,16,17,18,19,20,21,22,23,24,25]. Thus, due to the scarcity of available information about the ecology and biology of Neotropical Odonata, more studies and field observations are needed to be able to build up a “big picture” of the ecological and biological processes affecting these insects in tropical habitats [26].

Megapodagrionidae is a pantropical family of Odonata and includes the genus *Teinopodagrion* De Marmels, 2001, an endemic genus from the South American Yungas Mountain cloud forest, which extends from Venezuela to northwestern Argentina along the eastern slope of the Andes [27]. To our knowledge, population studies of *Teinopodagrion* species are nonexistent, but are necessary to know and understand the population biology and ecology of these tropical species, which are apparently present at very low densities in tropical ecosystems. In this paper, we present the results of a demographic and behavioural study of *Teinopodagrion oscillans* (Selys) from a population inhabiting a protected area of the Colombian Andes. The objectives of this research included: (i) to estimate population size, sex ratio, survival, and life expectancy, (ii) to study the area frequented by the adults by estimating the distance from the stream in which individuals of different age classes perch, and (iii) to describe patterns on colouration and general behaviour. 

## 2. Materials and Methods

### 2.1. Study Area 

The Club Naútico El Muña is a protected area located near Bogotá (Sesquilé, Cundinamarca) at an elevation of 2600 m at the freshwater reservoir Embalse del Tominé (4°50′9.80″ N and 73°55′0.70″ W). Although in this area, for more than 50 years, native flora and fauna species (e.g., Andean forests) have been protected; cosmopolitan species such as *Pennisetum clandestinum* Hochst. ex Chiov., *Eichhornia crassipes* (Mart.) Solms, *Acacia* sp., and *Eucalyptus* sp. can also be found. This protected area is very important because it harbours dragonfly and damselfly species that have disappeared from intervening surrounding areas [28]. The area includes a stone-bed first-order stream named Agua Clara, which originates in the Paramo ecosystem. The habitat of *T. oscillans* is a section of the Agua Clara stream, which is mixed with water from the Tominé dam, generating a mesotrophic system of slow currents surrounded by riparian vegetation and open woodland. 

### 2.2. Study Species

**Figure 1:**
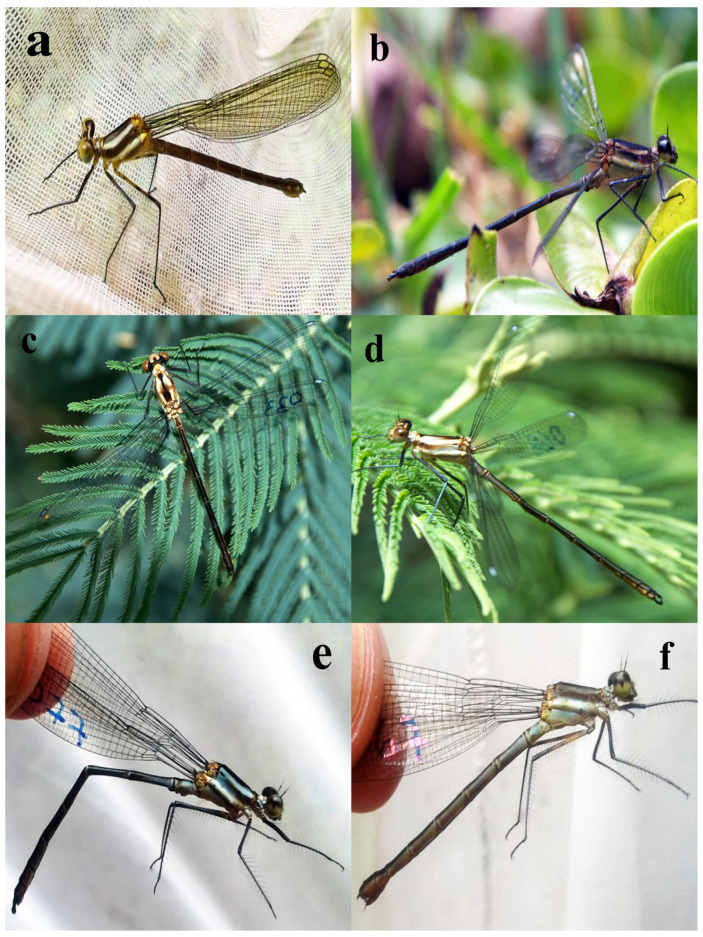
*Teinopodagrion oscillans* adult stages; (**a**) Teneral female; (**b**) sexually immature male; (**c**) sexually immature female; (**d**) sexually immature male; (**e**) sexually mature male; (**f**) sexually mature female.

*Teinopodagrion oscillans* is a medium-sized zygopteran (body length 38.04–45.94 mm; wing length 28–32.99 mm). This species has a bicoloured trapezoidal pterostigma, with sky-blue on the dorsal region and light brown in the ventral region, and is further characterised by long legs, the posterior femur reaching the abdominal segment 3 (Figure 1). Postocular spots are also sky-blue. Due to the development of pruinescence (i.e., waxy bloom developed on some species as they mature), older individuals develop a bluish-grey colouration on the thorax and abdominal segments 1–3 (Table 1, Figure 1). The abdomen is brown to black dorsally and light brown on the sides and ventral parts. There are also temperature-dependent colour changes, such as being more bluish, greenish, and brownish at lower temperatures. 

### 2.3. Data Collection 

Two transects of 500 m parallel to the dam shoreline were demarcated to sample both banks in the stream. Every individual observed along the transect was caught with an aerial net, marked with a fine-yipped sharpie on the right forewing using a four-digit code (at first capture), and its sex, colour (as estimator of age), and recapture data were recorded. Then, it was released at the site of capture within one minute [29]. A total of 270 h of sampling person effort was used in the study. Fieldwork was performed daily between 14 October 2020 and 1 January 2021. Both transects were sampled several times during each session, moving us 500 m upstream. However, no individuals were found in the periods 17–19 October and 9–10 November, days with unfavourable weather conditions. 

Following well-known methods to classify age in Odonata [30], adult individuals in this study were classified into three age categories, i.e., tenerals, those individuals with soft yellow wings lacking well-defined general body pigmentation; sexually immature, including individuals with entirely flexible wings, or flexible from the node to the tip, with a pale body colour; and sexually mature, those with hard and opaque, inflexible wings, and well-defined body colour. 

### 2.4. Behavioural Study 

The observations followed the focal animal temporal sampling technique [31]. Each session took place from 08:00 to 14:00 h (six hours daily) Colombian time (COT, UTC-5), divided into periods of 10 min per individual, in which the sequence and changes in behaviour were recorded. Ten minutes later, a different marked animal was observed. A total of 1800 min were used to collect observational data in the breeding area. We sorted our observations into the following categories: (i) perching; (ii) foraging; (iii) tandem; (iv) mating; (v) oviposition. Observations were performed with the naked eye or using 10 × 50 JHOPT™ binoculars (Brighton, CO, USA). 

### 2.5. Statistical Analyses 

The recapture histories of marked animals were analysed using Cormack-Jolly-Seber models with the software Mark 10.1 [32]. First, we tested the adjustment of the full time-dependent model by sex (model Phi(g*t) p(g*t), where phi is survival rate; p is detection probability; g indicates the group (males or females), and t refers to time (day of sampling)) by means of the program RELEASE. However, this model was not a good starting point because it did not fit the data (Test 2+ Test 3 by groups, χ^2^_347_ = 591.8, *p* < 0.001). Therefore, we divided adult males and females into teneral, immature and mature (as described above) and tested the fit of the new model, including six groups (three for each sex) and full time-dependence. This model fits the data (Test 2 + Test 3 by groups, χ^2^_403_ = 381.7, *p* = 0.771). In the second step, we estimated the variance inflation parameter (c-hat) by dividing the value of c-hat of the saturated model by the mean c-hat of 100 bootstrap simulations using Mark. The value obtained (c-hat = 1.0802) was used to correct estimations of standard errors. Models were ranked using their Akaike’s quasi-criterion of information, corrected for overdispersion (QAICc). Means are presented with the SE and (sample size). Statistical tests were calculated with xlStat 2021 (Addinsoft, Paris, France). 

## 3. Results

### 3.1. Biometry and Population Structure

In total, we marked 324 individuals, 167 males and 157 females, recapturing 276 (85%) and measuring age-specific recapture rate, lifespan, body size, and dispersal for each sex (Table 2). The proportion of animals that were recaptured was not significantly different by age and sex classes (contingence χ^2^_5_ = 9.8, *p* = 0.082). The number of recaptures was, on average 5.1 ± 0.20, including animals never resighted, and was not significantly affected by sex and age (ANOVA, F_3,320_ = 0.26, *p* = 0.855). The observed lifespan was 14.1 ± 0.59 days, with a maximum of 37 days, and, again, there were no significant differences by sex and age (F_3,320_ = 1.20, *p* = 0.311). Males were significantly longer than females (F_1,320_ = 8.21, *p* = 0.004; Table 2), and mature individuals were significantly longer than immatures for both sexes (F_2,320_ = 4.77, *p* < 0.009; Table 2). However, wing length was not significantly different between sexes and age groups (F_3,320_ = 1.27, *p* = 0.285). There was a significant correlation between body length and wing length, but surprisingly, wing length itself was not significantly different between sexes or age classes (r = 0.13, *p* = 0.017), and no significant correlation of either variable with the date of marking (correlation Body Length-Date, r = −0.07, *p* = 0.193; Wing-Date, r = 0.07, *p* = 0.198). Finally, the distance from the water was similar for all groups of individuals, averaging 2.7 ± 0.04 m from the stream (F_3,320_ = 0.39, *p* = 0.758). 

Considering the recapture histories of animals marked as teneral and immature, thanks to the high recapture rate of this population, it is possible to estimate the duration of the juvenile phases, i.e., the time elapsed from teneral to immature and from immature to mature (Table 2). The teneral phase lasted no more than one day because all individuals marked at this age that were recaptured the day after were already immature (N = 5 males and 4 females). The time elapsed to achieve mature colouration averaged 8.6 ± 0.40 (70) days for males and 9.3 ± 0.40 (75) days for females. This maturation period was similar for all individuals, irrespective of their sex and age at marking (F_2,142_ = 0.994, *p* = 0.372).

**Figure 2:**
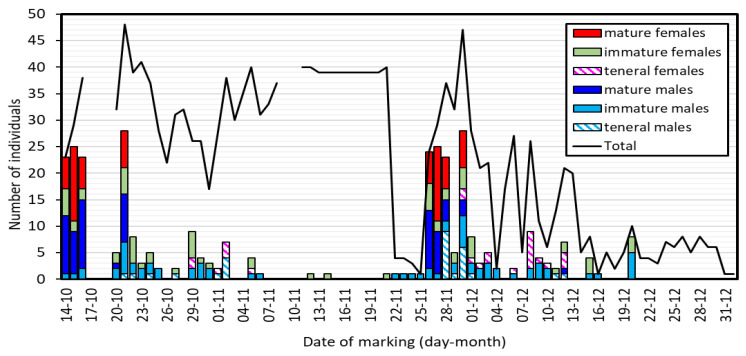
Daily variation in the number of new individuals found by age class (bars) and the total number found (including recaptures, Total). Note that the number of mature individuals was high at the start of the study and in the period 26–30 November, with a large gap with no mature individuals between these dates and after 30 November. No individuals were found during 17–19 October nor 9–10 November due to bad weather.

Over the period of study, the age composition of the population varied markedly (Figure 2). At the start of the fieldwork, most animals seen for the first time (87–92% of males and 55–88% of females in the period 14–16 October) were sexually mature. Many mature animals were found on 20 and 21 October, but then no new mature individuals were found until 26–30 November, when mature animals were again the majority of the new animals found (Figure 2). 

Considering also recaptured animals, the number of individuals found each sampling day was, on average, 22.2 ± 2.79, but with large variation, between 1 and 48 individuals (Figure 2, line Total). In the first period (14–21 November), the population was large (average of 34.5 individuals), but an abrupt diminution occurred after 22 November. The population recovered after 26 November, when many unmarked mature animals appeared. After that date, there was a continuous diminution of the population, which almost disappeared by the end of December.

### 3.2. Survival and Recapture Rates

**Figure 3:**
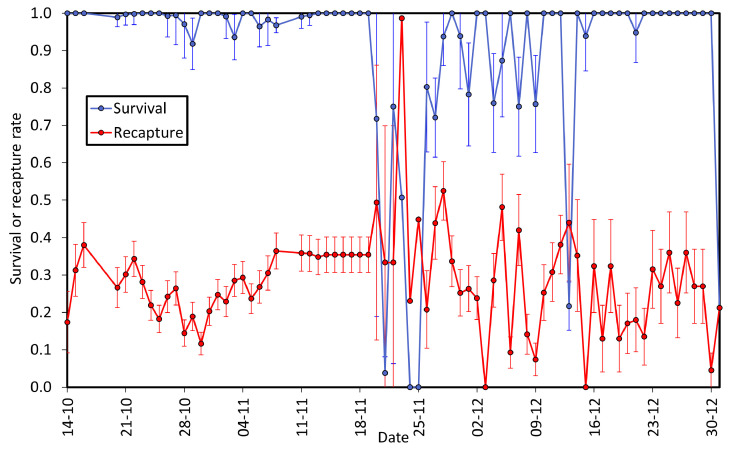
Estimates of daily survival and recapture rates (±SE) for the period of study, obtained from model Phi(t) p(t). Note the difference between the first period (14 October to 19 November) and the second (20 November to 1 January).

Supplementary Table S1 shows the results of fitting standard models to the recapture histories of marked animals. The most supported model is the time-dependent model for survival and recapture, with no effect of age and sex, model Phi(t) p(t). This model has an Akaike’s weight of 1, indicating that the rest of the models have no support and implies that there is evidence for daily variability of survival and recapture. Examination of the parameter estimates from this model (Figure 3) shows that after an initial period where estimates of recapture were 0.15–0.35, there was an erratic variation of this parameter starting on 20 November. On 22 November, only 2 animals were captured, compared to an average of 38 in the previous 15 days (Figure 3). The daily survival rate was about 1 before 20 November, and then also showed erratic behaviour but was again around 1 for the last days of fieldwork (Figure 3). This daily variation in survival and recapture is likely due to variable weather conditions. The average daily survival rate was 0.946 ± 0.0032, with a daily recapture rate of 0.269 ± 0.0072, estimated from the reduced model, Phi(.) p(.).

### 3.3. Behaviour

We compiled the following information based on observations of the behaviour of 324 *T. oscillans* individuals. Between 08:00 and 14:00 h individuals perched with open wings near water on macrophytes (36% of the observations), grass (7.6% of the observations; ≤10 cm high), human garbage carried by the current of the water into the protected area (7.6%), branches, stems and leaves of *Acacia* sp. (41.2%; range 0.5–12 m high; 2.71 ± 0.75 m) and even over us (7.6%). Teneral individuals remained perched with their wings closed. After two hours, they began to slowly open and close their wings. They perched with their body almost straight, in a vertical or horizontal position. Most of the specimens (80%) were found at the end of the stream, near the still water created by the dam. Our observations indicate high fidelity of the individuals to the perch site, remaining 0.5–122 min on the same perch. 

While teneral individuals and mature females remained motionless on the perch, mature males made frequent short flights (<5 s), returning quickly. From two to five individuals may share the same perch in branches of *Acacia* sp. Individuals were preferentially found perched on the tip of sunny branches at high temperatures (18–25 °C) but searched for sheltered places at lower temperatures (10–17 °C). Mature males were apparently not aggressive, but inspection flights to other damselflies and dragonflies were commonly observed, including approaches to other conspecifics (53.2% of the observations) and to individuals of coexisting species (29.3% of the observations), including *Mesamphiagrion laterale* (Selys), *Sympetrum gilvum* (Selys), *Rhionaeschna marchali* (Rambur), and *Rhionaeschna cornigera* (Brauer). Other short flights were predation events (17.5% of the observations). On several occasions, when two or more individuals shared a perch, if any of them changed to a nearby branch, one or more might follow. 

At the hottest moments, individuals perched with their bodies perpendicular to the branch. Mature males approached females, and if they were receptive, the tandem position was formed, mostly on macrophytes near the water. Only one pair in copula was observed because pairs in tandem flew away. We observed only three females ovipositing, all of them not accompanied by the male, but in the two first events, the male remained near the oviposition place, although obvious female guarding was not observed. The oviposition lasted for 11.23 ± 0.61 min (N = 3), with the females ovipositing by abdomen submersion.

## 4. Discussion

There is little biological information about most genera in the Megapodagrionidae family, and *Teinopodagrion* is not an exception. Our study is the first to compile demographic information for a species in this genus and yielded some unexpected results. First, we were able to recapture 85% of the animals marked, a value rarely achieved with odonates. Second, males and females were very similar in all demographic parameters, which is unusual. Third, the population studied showed two clear peaks of abundance, separated by six weeks. Finally, both recapture and survival rates showed strong daily variation (Figure 3).

*Teinopodagrion oscillans* show little sexual dimorphism in body colouration, as is typical of the species of the genus (for instance, see the pictures of *Teinopodagrion meridionale* De Marmels in [33]). Males are slightly larger than females, but their wing lengths are not different (Table 2), implying that, to maintain flight performance, wing morphology should be sexually dimorphic, given the different weights of male and female damselflies [34]. Body and wing lengths were not correlated with the date of marking. In temperate species, a decrease in body size is frequently observed during the season [35,36] due to time constraints and the effect of rising temperatures, and this is a general pattern in aquatic arthropods [37]. However, in tropical environments, where seasonal limitations are scarce, body size is not expected to correlate with date [34,38,39,40]. 

In a recent review of mark-recapture studies with odonates, Sanmartín-Villar and Cordero-Rivera [12] found that, in general, males and females differ in their recapture rates but not in survival, provided that the study period is long enough to produce unbiased estimates. These authors also found that age at marking affected recapture rates and that males are recaptured in a higher proportion than females. In our population, however, both sexes and all age groups showed similar recapture rates and observed lifespan (Table 2). *Teinopodagrion oscillans* individuals show high site fidelity and concentrate near the stream, being found on average at a distance lower than 3 m from the water. This behaviour explains the high recapture rates achieved during this study, similar to the case of another damselfly living in tropical streams [41].

The average time between marking and last recapture was two weeks and was not significantly different between sexes. In general, observed lifespan is higher in males, and this has been explained by their behaviour because males usually remain closer to the water than females, and, as a consequence, male recapture rates are higher (e.g., [42]). For instance, in *Hypolestes trinitatis* (Gundlach), an endemic damselfly from Cuba, which has many ecological similarities with *T. oscillans*, recapture rates were much higher in males [43]. The estimated survival rate of *T. oscillans*, 0.946, is one of the highest ever estimated for adult odonates, identical to the male survival rate estimated for *H. trinitatis* [43] and similar to other species with highly territorial behaviour [12]. Some Calopterygids, Polythorids, Lestids and Thaumatoneurids have daily survival rates above 0.96 (e.g., [44,45]), which translate into expected longevities of around 24 days, using the formula of Cook et al. [45]. In our dataset, the expected longevity is 18 days, and the maximum observed longevity was 37 days, a value that is nevertheless lower than the maximum longevity of *Calopteryx* Leach, which is about 40 days (e.g., [46]).

The first nine days of adult life include the sexual maturation period in *T. oscillans*. This means that the net reproductive life for those animals surviving until maturation is, on average, about one week (Table 2). Sexual maturation is needed in all odonates because females do not have mature eggs when they emerge from the exuvia, and males do not have functional sperm. In general, the pre-reproductive period is longer in females than in males (reviewed by [47]), but in our population, this was not the case. In tropical damselflies, the maturation period can be very long, even more than one month in giant damselflies [48], but species with similar body size as *T. oscillans* have shorter maturation periods. For instance, male *Polythore mutata* (McLachlan) need 12.6 days to mature [49], and *Palaemnema desiderata* Selys and *Palaemnema paulitoyaca* Calvert 7–10 days [50]. 

The frequency of reproductive events was low, and this could be due to some reproductive events not coinciding with the time of our study [51] or they took place in locations difficult to access by the observers, e.g., elevated twigs in the vegetation or quiet sites with high species richness of plants [18].

One of the most unexpected results of our study is the large daily variation in the appearance of mature individuals (Figure 2). Apparently, two cohorts matured with a separation of about six weeks between them, also producing a clear difference in population size between the first and the second half of the study (Figure 2). One possible explanation for this is that these two cohorts represent two generations [52,53]. However, the time elapsed seems too short for larval development. Population size estimates for damselflies from tropical streams are scarce. In a study with a duration of two months of a population of *H. trinitatis*, male and female abundance were estimated to show little daily variation, with slow increases or decreases, but not sudden changes [41]. Similar patterns emerge from a population study of *Argia chelata* Calvert [54] in Costa Rica and two populations of *Heteragrion cooki* Daigle and Tennessen from Ecuador [16]. However, a mark-recapture study of the damselfly *Ischnura pumilio* (Charpentier) in Spain also showed the appearance of two population peaks of mature adults, in this case with a separation of 10 days [55], suggesting immigration from another population. 

Growth rates, timing of emergence, and flight seasons can show inter-year variation within species in the same habitat [56]. For several Odonata species, it is known that the size of the last instar larvae is a complex function of temperature, time of emergence, and species identity. Cothran and Thorp [57], for example, found that in the same habitat, individuals of the same species were smaller at the warm micro-habitats, while the largest animals emerged from the cooler micro-habitats. Temperature within lentic freshwater habitats can vary broadly among microhabitats throughout the day and over time [58]. In our study area, something similar probably happened with temperature due to the variety of aquatic microhabitats provided by variations in water depth, number and type of macrophytes, and turbidity caused by the mixing of reservoir water with that of the stream. Although we did not measure these aspects, it is plausible that morphological and behavioural responses can be adapted to the varying conditions of the microhabitat where the animals live. 

Our study was also unique because daily recapture and survival rates were clearly variable during the period of fieldwork (Figure 3) despite similar sampling efforts. Between 14 October and 19 November, we marked a total of 155 individuals, almost half of the total marked in the whole study, and all of them disappeared by 22 November, producing an abrupt change in population size (Figure 3). Odonate adults strongly depend on ambient temperature to carry out their daily activities [59,60]. The protected area, Club Náutico El Muña, is in an area where temperatures vary between −2 and 25 °C throughout the day and over the days [61]. Therefore, it is plausible that the number of individuals may change rapidly between days in microhabitats due to these temperature variations. We observed that individuals of *T. oscillans* in this area are indiscriminate when selecting their perch, but on very cold days, they stayed on high branches (10–12 m high), where the individuals and their numbers were difficult to track. The influence of microhabitats on *T. oscillans* seems remarkable, because in addition to the low temperatures, the ecosystem was flooded by the water of the reservoir due to the rains in February 2021, strongly reducing the number of microhabitats available for oviposition and possibly the growth of larvae. 

On the other hand, upstream or downstream movements are common in odonates [62], for instance, to explore the area, to compensate for downstream larval drift [63], and to choose sites where competition between adults, larvae, or during emergence is lower [64]. Although we do not know the magnitude of these distances, individuals of *T. oscillans* could travel up to 2 km upstream and be able to return, as has been reported for *Calopteryx haemorrhoidalis* (Vander Linden), a damselfly of similar size (45–48 mm) [65], whose adults have also been observed to move from one stream drying up to another that was permanent. Our study only included 0.5 km of a minimum of 10 km that the Agua Clara Creek runs into the Páramo, so more studies will be needed to test whether the abundance of *T. oscillans* could depend on migrations to and from other microhabitats in this creek.

Odonates are indicators of ecosystem health and the effect of environmental changes [66]. The information from this study allowed us to recognise a population of *T. oscillans* with hundreds of individuals and a high life expectancy. The protected area Club Náutico El Muña provides a heterogeneous plant structure and a high diversity of insects and other organisms that allow activities (e.g., perching, shelter, feeding and reproduction) for the survival of the population. It is likely that these conditions are benefiting many other species, so it is important that the protection processes in this reserve continue to prevent the impact of human activities from negatively altering this habitat and affecting the species that inhabit it.

Our study provides crucial information for the understanding of basic population aspects of *T. oscillans*, a species categorised as Least Concern, because it has been reported for two protected areas, one in Colombia [67] and the other in Venezuela [68]. However, our findings support the idea that it is important to know how populations are affected by local conditions to allow evidence-based categorisations and conservation decisions. Because survival and the probability of recapture were time-dependent, environmental temperature is a likely factor that alters the presence and size of this population, which we will continue to monitor to try to understand the dynamics that in the medium and long term affect the conservation of this species in habitats of the Andean region. 

## Figures and Tables

**Table 1:** Colour in different body regions of *T. oscillans* at different ages. PS, Postocular spots; MSP, Mesepisternum; MES, Mesepimeron; MET, Metepisternum; MTP, Metepimeron; S1–3, Abdominal segments 1–3 dorsal and lateral regions; W, Wings; Pt, Pterostigma.

Age	Teneral	Sexually Immature	Sexually Mature
PS	Yellow-Light Brown	Light blue	Light blue
MSP	1 longitudinal dark brown stripe and 1 longitudinal yellow stripe	Light blue (80%) and Light brown (20%)	1 longitudinal black stripe and 1 longitudinal light blue stripe
MES	1 longitudinal brown stripe	Light blue (60%) and Light brown (40%)	Light blue (80%) and Light brown (20%)
MET	1 longitudinal yellow stripe	Light blue (90%) and Light brown (10%)	Light blue
MTP	Yellow-Light brown	Light blue (80%) and Light brown (20%)	Light blue
S1–3	Yellow-Light brown	Light blue (50%) and Light brown (50%)	Light blue (80%) and Light brown (20%)
W	Yellow	Hyaline	Hyaline
Pt	Yellow-Light brown	Light blue	Light blue

**Table 2:** Summary statistics of the population of *Teinopodagrion oscillans* marked in this study. Recaptures indicate the number of days in which the individual was found, and lifespan refers to the number of days between marking and the last recapture. Maturation indicates the time in days to achieve mature colouration, only for animals marked as teneral or immature. Distance indicates the distance to the water in m at the moment of first capture. Body size measurements in mm. Sample size is the number of marked specimens, except indicated otherwise. A, Age; T, Teneral; I, Immature; M, Mature; Mk, Marked individuals; R, Recaptured (%) individuals; SE, standard error of mean; N, sample size.

				Recaptures		Lifespan		Maturation		Body Length		Wing Length		Distance	
Sex	A	Mk	R	Mean	SE	Mean	SE	Mean	SE (N)	Mean	SE	Mean	SE	Mean	SE
Male	T	28	25 (89.3)	5.18	0.55	12.75	1.56	9.43	0.57 (21)	41.86	0.31	30.69	0.30	2.82	0.15
	I	70	53 (75.7)	4.64	0.41	12.87	1.28	8.16	0.47 (49)	41.50	0.23	30.51	0.16	2.64	0.08
	M	69	48 (70.0)	5.70	0.48	16.12	1.38	-	-	42.01	0.25	30.40	0.17	2.71	0.08
Female	T	26	24 (92.3)	4.31	0.51	10.46	1.26	8.95	0.52 (29)	40.89	0.29	30.34	0.28	2.77	0.13
	I	65	51 (78.5)	5.80	0.46	16.08	1.29	9.22	0.48 (55)	40.79	0.23	30.14	0.16	2.69	0.10
	M	66	46 (70.0)	4.77	0.43	13.39	1.43	-	-	41.68	0.22	30.92	0.16	2.71	0.10

## Data Availability

The data presented in this study are available on request from the corresponding author (odonata17@hotmail.com).

## Supplementary Materials

The following supporting information can be downloaded at: https://www.mdpi.com/article/10.3390/insects15020125/s1, Table S1: Results of model selection for T. oscillans. Survival rate is denoted by Phi and recapture probability by p. The group is sex (g) and time variation is indicated by t. The best model (in bold) includes variation due to time of recapture and survival.
