# Supplementary material for: Demography and Behaviour of Teinopodagrion oscillans (Odonata: Megapodagrionidae) in a Protected Area of the Colombian Andean Region
Source: Insects. 2024 Feb 9;15(2):125. doi: 10.3390/insects15020125 (PMC10889271; doi:10.3390/insects15020125)
Supplement: Supplementary file 1 [file insects-15-00125-s001.zip › insects-2846719-supplementary.pdf]

Supplementary material

Table S1. Results of model selection for *T. oscillans*. Survival rate is denoted by *Phi* and recapture probability by *p*. The group is sex (*g*) and time variation is indicated by *t*. The best model (in bold) includes variation due to time of recapture and survival.

Model	QAICc	Delta QAICc	AICc Weights	Model Likelihood	Num. Par	QDeviance
**{Phi(t) p(t)}**	**5912.62**	**0.00**	**1**	**1**	**146**	**4790.41**
{Phi(t) p(.)}	5938.56	25.95	0	0	75	4979.50
{Phi(t) p(g)}	5944.01	31.39	0	0	80	4973.94
{Phi(g*t) p(g*t)}	5981.22	68.61	0	0	337	4333.29
{Phi(.) p(t)}	6230.29	317.68	0	0	75	5271.23
{Phi(g) p(t)}	6237.26	324.65	0	0	80	5267.19
{Phi(.) p(.)}	6426.83	514.22	0	0	2	5620.96
{Phi(g) p(.)}	6431.85	519.24	0	0	7	5615.91
{Phi(.) p(g)}	6433.26	520.64	0	0	7	5617.32
{Phi(g) p(g)}	6438.21	525.60	0	0	12	5612.15
{Phi(t) p(g*t)}	6591.69	679.08	0	0	482	4430.90
{Phi(g*t) p(.)}	6652.85	740.24	0	0	382	4858.20
{Phi(g*t) p(g)}	6665.42	752.81	0	0	387	4853.83
{Phi(g*t) p(t)}	6692.13	779.51	0	0	449	4658.84
{Phi(.) p(g*t)}	6838.59	925.97	0	0	429	4879.25
{Phi(g) p(g*t)}	6848.43	935.81	0	0	434	4870.84
